# Human platelet lysate to substitute fetal bovine serum in hMSC expansion for translational applications: a systematic review

**DOI:** 10.1186/s12967-020-02489-4

**Published:** 2020-09-15

**Authors:** M. Guiotto, W. Raffoul, A. M. Hart, M. O. Riehle, P. G. di Summa

**Affiliations:** 1 Department of Plastic, Reconstructive and Hand Surgery, Centre Hospitalier Universitaire Vaudois (CHUV), Lausanne, Switzerland; 2 Centre for the Cellular Microenvironment, University of Glasgow, Glasgow, UK; 3 Canniesburn Plastic Surgery Unit, Glasgow Royal Infirmary, Glasgow, UK

**Keywords:** Human platelet lysate, Foetal bovine serum, Mesenchymal stem cells, Adipose derived stem cells, Cell therapy

## Abstract

**Background:**

Foetal bovine serum (FBS), is the most commonly used culture medium additive for in vitro cultures, despite its undefined composition, its potential immunogenicity and possible prion/zoonotic transmission. For these reasons, significant efforts have been targeted at finding a substitute, such as serum free-media or human platelet-lysates (hPL). Our aim is to critically appraise the state-of-art for hPL in the published literature, comparing its impact with FBS.

**Materials and methods:**

In June 2019 a systematic search of the entire Web of Science, Medline and PubMed database was performed with the following search terms: (mesenchymal stem cells) AND (fetal bovine serum OR fetal bovine calf) AND (human platelet lysate). Excluded from this search were review articles that were published before 2005, manuscripts in which mesenchymal stem cells (MSCs) were not from human sources, and when the FBS controls were missing.

**Results:**

Based on our search algorithm, 56 papers were selected. A review of these papers indicated that hMSCs cultured with hPL showed a spindle-shaped elongated morphology, had higher proliferation indexes, similar cluster of differentiation (CD) markers and no significant variation in differentiation lineage (osteocyte, adipocyte, and chondrocyte) compared to those cultured with FBS. Main sources of primary hMSCs were either fat tissue or bone marrow; in a few studies cells isolated from alternative sources showed no relevant difference in their response.

**Conclusion:**

Despite the difference in medium choice and a lack of standardization of hPL manufacturing, the majority of publications support that hPL was at least as effective as FBS in promoting adhesion, survival and proliferation of hMSCs. We conclude that hPL should be considered a viable alternative to FBS in hMSCs culture—especially with a view for their clinical use.

The word “mesenchymal stem cells” or MSCs and “stromal cells” are here used as synonym despite the recent distinction between the tissue-specific (e.g. adipose, bone, cartilage, …) stem/progenitor cells and the old generic term mesenchymal stem cells [1].

## Background

The application of cellular therapies is growing enormously in a wide range of medical fields, often using human mesenchymal stromal/stem cells (hMSCs).

In the clinic hMSCs are currently used for bone marrow transplantation, to treat bone or cartilage defects, myocardial infarction and to manage graft versus host disease. Potentially for regenerative purposes, hMSCs could also be applied in combination with tissue engineering strategies to treat musculoskeletal and neurological disorders [2].

hMSCs are non-hematopoietic progenitors, with an ability to differentiate along mesenchymal and non-mesenchymal lines, such as adipose, chondrocyte or osteoblast lineages. Bone marrow, umbilical cord blood, dental pulp and adipose tissue are all potential sources for autologous hMSCs in stem cell-based therapies. However, bone marrow (BM-hMSCs) and adipose derived mesenchymal stromal/stem cells (ADSCs) are more popular.

Isolation of BM-hMSCs is a complex and painful process for the patient, with a low cell yield, while ADSCs extraction by liposuction is a less invasive procedure with minimal patient discomfort. Moreover, adipose tissue has a 500 times higher number of stem cells than bone marrow per unit of tissue (weight/volume), therefore it has recently been recognized as the source of choice for hMSCs [3].

Despite that, the clinical applications of hMSCs is hindered by their availability, because the number of cells that can be safely isolated per unit tissue volume is limited. Thus, hMSCs need to be expanded and may have to be differentiated ex vivo before their therapeutic application, requiring in vitro culture. The entire process (isolation, in vitro culture and eventual differentiation) must respect the principles of good manufacturing practice (GMP) in order to minimize risks and maximize the benefits of cell therapy [4, 5].

### Foetal bovine serum and human platelet lysate

Foetal bovine serum (FBS), also called foetal calf serum, represents the most common serum additive for in vitro usage, which supports adhesion, growth and proliferation of a wide spectrum of different cells. FBS usually is extracted from foetal bovine blood, being collected after slaughter of pregnant cows under sterile conditions. After clotting, centrifugation steps are carried out to separate its cellular components, before filtration steps (normally 100 nm pore size) are applied to remove potential bacterial and viral contaminations. FBS is well known for its cost effectiveness and its richness in adhesion molecules, growth factors, micronutrients and hormones which promote attachment, growth and proliferation of mammalian cells.

In addition, FBS enables expansion of human cell cultures in vitro and supports trilineage differentiation potential (osteogenic, adipogenic and chondrogenic) of hMSCs. Therefore, it has, until now, been the media supplement of choice in a wide range of cell culture protocols [6].

Despite that, the composition of FBS medium additive is not completely determined, with a wide heterogeneity among samples [6]. Furthermore, when used in a translational setting, anaphylactoid reactions and the risk of zoonoses transmission were reported [7, 8]. These reports suggest that using FBS is, in essence, not compliant with the principles of GMP, because it may affect the safety and efficacy of cell therapy [9–11].

For these reasons, significant efforts are targeted at finding a substitute for animal serum such as serum free media or platelet derivates [12]. Alternatives to FBS and serum free media, that could help for the translation of cell therapy to the clinic, are human serum (hS), platelet-rich plasma (PRP) and human platelet lysate (hPL). When allogeneic hS is implemented in cell expansion hMSCs proliferation rate is reduced and the time it takes for cells to reach confluence extended compared to the standard FBS cultures [13]. In addition, the concentration of growth factors seems to be limited in both hS and FBS, and some authors found that the debris, that forms during PRP production, could slow down cell expansion [6, 14]. However, the relative advantages of these different culture media additives are still widely debated and recent studies showed that particularly hPL could be a valid substitute for FBS, retaining stem cell phenotype (positive expression of CD (cluster of differentiation) CD73, CD90, CD105, but negative for CD34 and CD45) and multilineage differentiation capacity (osteogenic, adipogenic and chondrogenic) of hMSCs [15–17].

hPL can be easily prepared using at least three different manufacturing methods, such as plateletpheresis, “buffy coat” (BC) or platelet-rich plasma (PRP). Releasing the growth factors from the platelets represents the key point in hPL production: again, four different methods are available to attain platelet lysis: repeated freeze/thaw (FT) cycles, direct platelet activation, sonification alone or in combination with thermolysis and solvent/detergent (S/D) treatment.

How these means of preparation influence the biochemical and functional properties of hPL, has not yet been sufficiently investigated. hPL has the higher concentration of growth factors (GFs), such as platelet-derived growth factors (PDGF-AB), basic fibroblast growth factor (bFGF), transforming growth factor beta (TGF-ß1), insulin-like growth factor 1 (IGF-1) and vascular endothelial growth factor (VEGF), than any other cell culture supplements including PRP and FBS: this may be the reason why the majority of recent reports agree that hPL supports cell expansion to a higher degree, compared to FBS, hS and PRP [11, 13, 18–20]. Being a human blood derivate, allogeneic hPL requires to be tested for HIV, hepatitis B, C and other potential viral infections [6, 13, 21].

From a physiological perspective, platelets contain a great variety of growth factors, cytokines, proteins and factors that support clotting. When platelets are lysed, they release their content consisting of albumin, folate, vitamin B12, glucose, triglycerides and cholesterol (less concentrated in comparison to FBS), immunoglobulins (mainly IgG, in higher concentration compared to FBS) and other proteins that also contribute to balance media colloid pressure. In addition, platelets contain numerous growth factors, mainly TGF-ß, platelet-derived growth factors (PDGF-AB, PDGF-AA, PDGF-BB), IGF-1, brain-derived growth factor (BDNF), epidermal growth factor (EGF), VEGF, bFGF and hepatocyte growth factor (HGF) that are suitable to sustain growth of a wide range of cell types [22, 23].

Finally, two different types of hPL (autologous and allogeneic), related to the source, were proposed as suitable FBS alternatives. The risk of contamination and adverse immune reactions is lower in autologous rather than allogeneic material, but the volume of hPL that can be produced from autologous blood is not sufficient for clinical use. Autologous hPL also manifests a challenge due to a lack of standardization, related to donor patient’s heterogeneity which may lead to variations in biological effectiveness [24–26].

In contrast, derivation of allogeneic hPL can be automatized, and standardized in terms of tests and contents, which means that it could be a good candidate to substitute FBS in translational regenerative medicine [27].

### Preparation techniques of hPL

The methods applied to prepare hPL could possibly change its biochemical and functional properties, although if and how has not been evaluated yet in depth. Pooled allogenic platelets can be obtained from whole blood (e.g. sourced from transfusion banks) by three different separation protocols: buffy coat (BC), platelet-rich plasma (PRP) or plateletpheresis technology [27]. In the first technique the whole blood sample is firstly centrifuged (3000*g* for 5 min at 21–22 °C with a validated acceleration and deceleration curve) to separate cell components (buffy coat) from cell-free plasma which forms the top layer of the suspension. Then the buffy coat of four units (450–525 ml per unit) are mixed with a specific amount of plasma, followed by a second centrifugation and filtration steps (2μm pore size) in order to remove leukocytes [2, 28]. In the second method (PRP) four or five whole blood units are pooled and centrifuged (1000*g* for 10 min at 21–22 °C) to separate the blood cells from the upper layer consisting of platelets mixed with plasma. In plateletpheresis, blood, taken directly from a donor, and processed by an apheresis machine that uses centrifugation to remove a selected component from the blood and returns the remainder to the donor.

The resulting platelet concentrates are stored for 5–7 days at 22 °C under agitation [29]. To avoid bacterial contamination, platelet units should be stored at 22 °C for up to a maximum of 7 days from the time of donation. If they are not used with a clinical purpose, they can be frozen and subsequently be used for hPL production. However, no data exist that clearly state the maximum period of time, beyond 7 days, for which platelet units can still be used to obtain an efficient and safe hPL product [30].

The next step of preparation consists of the release of the growth factors from the platelets.

- Repeated freeze/thaw cycles: the commonest and easiest to implement technique consists of cycling (one to five times) between freezing at −30 or −80 °C and thawing at 37 °C to induce fragmentation of the platelets [19, 23].
- Direct platelet activation: adding a calcium salt (commonly CaCl_2_) which activates the endogenous thrombin cascade and leads to platelets lysis. Alternatively, human or recombinant thrombin can also be used to induce lysis [31, 32].
- Sonification alone or in combination with freeze/thaw cycles: up to 30 min at a frequency of 20 kHz releases platelet granules rapidly [33, 34].
- Solvent/detergent (S/D) treatment: it induces both platelet lysis and inactivates lipid-enveloped virus [27].

A single unit of hPL is obtained by mixing lysates of different origin. A further centrifugation step is needed to deplete the hPL off platelet fragments, then it can be stored at − 30 to − 80 °C until use [2].

Rauch et al. stated that hPL can be conserved at − 20 °C for at least 5 months, maintaining the same concentration of growth factors, especially EGF [26]. Moreover, hPL can be stored at 4 °C for 4 weeks without changing its efficacy [31], while other authors recently have reported a longer time period (2 years) over which it maintained its properties, if stored at − 80 °C [35].

### The aim

Our aim is to critically appraise and summarize the state of art in the published literature on how hPL supports in vitro cell culture of hMSCs in comparison to FBS. We collated the currently available evidence and envisage that this will be a helpful guidance in future experimental studies, related to the use of hPL and on its use as a supplement for translational applications.

## Materials and methods

In February 2019 (repeated in June 2019), a systematic review of the entire Web of Science (https://clarivate.com/webofsciencegroup/solutions/web-of-science/), Medline (https://www.nlm.nih.gov/bsd/medline.html) and PubMed (https://pubmed.ncbi.nlm.nih.gov) database was performed with the following search terms: (mesenchymal stem cells) AND (fetal* bovine serum OR fetal* bovine calf) AND (human platelet lysate). Secondly ADSCs AND (FBS OR FBC) AND hPL. Finally, bibliography references were analysed and included if pertinent.

**Fig. 1:**
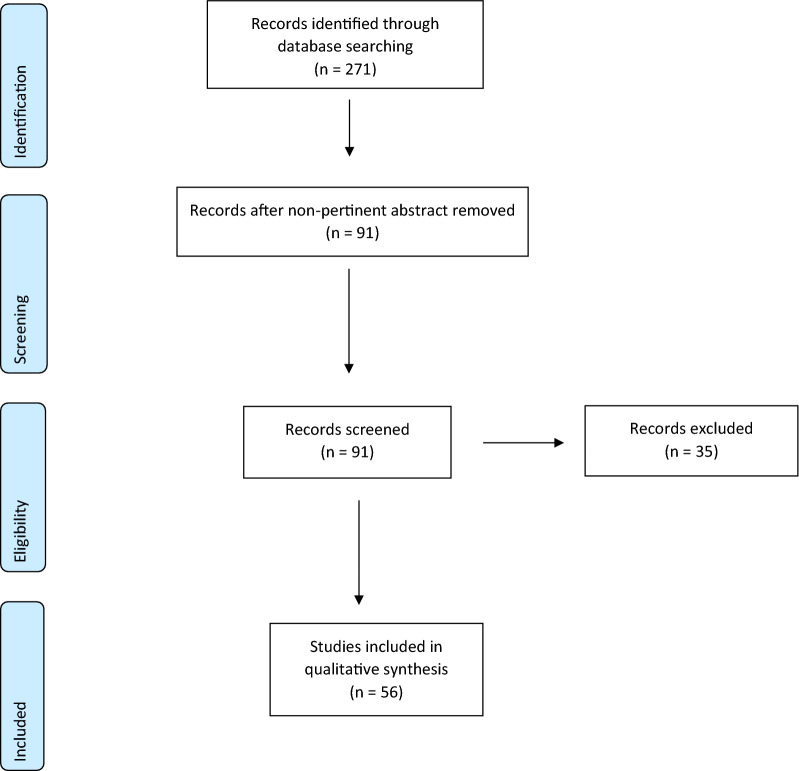
PRISMA flow chart selection process

All article types, except reviews, that were published in English between 2005 and 2019 were considered without restrictions. Other exclusion criteria were MSCs not sourced from human material and papers where FBS/FCS controls were lacking. All publications were screened manually, and the data extracted according to predetermined criteria. The flow chart of article selection follows the Preferred Reporting Items for Systematic Reviews and Meta-Analyses (PRISMA) statement (Fig. 1) [36].

Apart from bibliometric information, the following data was extracted and tabulated for each article (Table 1): source of MSCs, concentration of FBS, concentration of hPL, method of platelet extraction, hPL source (autologous/allogeneic), procedure to create platelet lysate, feeding schedule, growth index (proliferation rate, cell count, cell population doubling time or generation time or CFU-U number or cell viability), cell morphology, immunophenotype, MSCs differentiation potential.

**Table 1:** Selected publications between 2005 and 2019 in Web of Science database according to the following search terms: (mesenchymal stem cells) AND (fetal bovine serum OR fetal bovine calf) AND (human platelet lysate), its acronyms and complete bibliography checking

Author	Type of cell	% Additive	hPL manufacturing	Type of lysis/Inactivation	Filter	Diff. Line	T. Feeding	MO	P.I.	CD
(Kong et al. 2019)	hUMC-MSCs	10% FBS, 10% hPL, 10% HS	I	N.M.	N.M	1	3–4 days	2	2	1
(Barro et al. 2019)	BM-MSCs	FBS, hPL, i-hPL	Aph	SD + UV, FT	0.22/0.1 µm	3	3–4 days	1	1	1
(Lensch et al. 2018)	ASC-MSCs	FBS 10%, xeno free media	I	N.M.	N.M	1	3–4 days	1	1	1
(Haack-Sørensen et al. 2018)	ASC-MSCs	10% FBS, 5% hPL	I	N.M.	N.M	1	N.M.	3	1	1
(Dessels et al. 2019)	ASC-MSCs	FBS 10%, 10%, 5% hPL	BC	FT	0.22 µm	0	3–4 days	1	1	1
(Becherucci et al. 2018)	BM-MSCs	FBS 10%, hPL 5%	BC	FT	0.45/0.22 µm	1	3–4 days	1	1	1
(Chen et al. 2019)	hUMC-MSCs	10% FBS, 6.55%(FTPL), 6.50%(GBPL), 7.50% (SDPL), 6.72% GBHPL	Aph	SD + Ca, FT	0.22 µm	A−/B+	3–4 days	1	1	1
(Ren et al. 2018)	BM-MSCs	20% FBS, 10% HPGF-C18	Aph	SD	0.2 µm	0	3–4 days	2	2	1
(Kandoi et al. 2018)	hUMC-MSCs	20% FBS, 10%, 5% hPL	N.M.	FT	N.M	1	3–4 days	2	1	1
(Phetfong et al. 2017)	ASC-MSCs	10% FBS, 10% hPL, 10% hPL + hPlasma	N.M.	FT	0.2 µm	B	1 day	1	1	1
(Søndergaard et al. 2017)	ASC-MSCs	10% FBS, 5% hPL	I	N.M.	N.M	0	3–4 days	1	1	1
(Mangum et al. 2017)	ASC-MSCs	FBS 10%, hPL 10%	I	N.M.	N.M	A−/B+	N.M.	3	1	2
(Bernardi et al. 2017)	BM-MSCs	10% FBS, 10%, 7.5%, 5% hPL, 10% 7.5% 5% PR-SRGF	Aph	Ca, FT	70 µm	1	3–4 days	3	2	1
(Pierce et al. 2017)	BM-MSCs	10% FBS, 10% PL-S, 10% PL-P	PRP	Ca	N.M	0	3–4 days	3	1	2
(Fernandez-Rebollo et al. 2017)	BM-MSCs	10%FBS, 10% hPL	Aph	FT	0.2 µm	1	N.M.	1	1	1
(Matthyssen et al. 2017)	hC-MSCs	FBS, hPL, hS 2.5%, 5%, 10%	I	N.M.	N.M	1	N.M.	1	1	1
(Viau et al. 2017)	BM-MSCs	2-15% FBS + bFGF, hPL, PR-hPL	BC	UV, FT	N.M	1	3–4 days	3	1	1
(Riis et al. 2016)	ASC-MSCs	10% FBS, 10%, 5% hPL	I	N.M.	N.M	0	3–4 days	4	1	2
(Escobar and Chaparro, 2016)	ASC-MSCs	FBS 10%, hPL 5%	BC	FT	0.22 µm	A−/B+	3–4 days	2	1	1
(Heathman et al. 2016)	BM-MSCs	10% FBS, 10% PL	I	N.M.	N.M	1	3–4 days	3	1	1
(Mohammadi et al. 2016)	hUMC-MSCs	10% FBS, 2%, 5%, 7%, 10% PL	PRP	N.M.	N.M	1	3–4 days	1	1	2
(Shirzad et al. 2017)	hUMC-MSCs	10% FBS, 10%, 5% UCB-PL, PB-PL	PRP	FT	N.M	3	3–4 days	2	1	2
(Suri et al. 2016)	hC-MSCs	10% FBS, 10% PHPL, 10% hPL	Aph	FT	N.M	0	N.M.	2	2	1
(Juhl et al. 2016)	ASC-MSCs and BMSCs	10% FBS, 5% PLTMax, 5% hPLS, 5% hPLSP	I	N.M.	N.M	1	3–4 days	1	1	1
(Muraglia et al. 2015)	BMSCs, ASCs, UC-MSCs, T-lymph	10% FCS, 5% PPP, hPL	PRP	FT	N.M	1	3–4 days	3	1	1
(Wagner et al. 2015)	ASC-MSCs and hF	10% FBS, 5% hPL	Aph	FT	0.2 µm	1	N.M.	3	1	3
(Riordan et al. 2015)	hUMC-MSCs	10% FBS, 5%, 7.5%, 10% XcytePLUS-hPL	N.M.	N.M.	N.M	1	N.M.	2	1	1
(Castiglia et al. 2014)	BM-MSCs	10% FBS, 10% hPL, 10% iHPL	BC	UV	0.2 µm	1	3–4 days	1	2	1
Trojahn Kølle et al. 2013)	ASC-MSCs	10% FBS, 10% hPL	PRP	FT	0.2 µm	1	3–4 days	1	1	1
(Witzeneder et al. 2013)	ASC-MSCs and hF	10% FBS, 5% hPLp, 5% hPLn, 10% huS	BC	FT	N.M	1	3–4 days	2	1	3
(Kinzebach and Bieback, 2013)	ASC-MSCs and BMSC	10%, 7.5%, 5%, 2.5% FBS, pHPL, tPRP	BC	Thr, FT	0.45 µm	A−/B+	N.M.	3	1	3
(Mojica-Henshaw et al. 2013)	MSCs	10%FBS, 10% hPLp, 10% hPLs	PRP	Ca, FT	0.22 µm	A	3–4 days	3	1	1
(Bernardi et al. 2013)	BM-MSCs	10% FBS, 10% PLT-FT-Ly, 7.5%, 5%, 2.5% PLT	PRP	Son, FT	70 µm	1	3–4 days	3	1	1
(Shanskii et al. 2013)	ASC-MSCs	10/0, 7.5/2.5, 5/5, 2.5/7.5, 0/10% FBS, hPL	N.M.	FT	N.M	0	N.M.	3	1	3
(Griffiths et al. 2013)	BM-MSCs	16% FBS, 2%, 5%, 10% hPL	PRP	FT	N.M	1	N.M.	1	1	1
(Ben Azouna et al. 2012)	BM-MSCs	10%FBS, 10% FBS + 5% hPL, 10% hPL, 5% hPL	Aph	FT	N.M	AB−	3–4 days	1	1	1
(Gottipamula et al. 2012)	BM-MSCs	10% FBS, 10% hPL	N.M.	FT	N.M	3	N.M.	1	1	1
(Fekete et al. 2012)	BM-MSCs	20% FBS, 10% hPL	BC	UV, FT	0.45/0.22 µm	1	3–4 days	3	2	1
(Schallmoser and Strunk, 2013)	hUMC-MSCs	10% FBS, 2.5, 5, 10% pHPL	BC, Aph	FT	0.22 µm	0	N.M.	3	1	3
(Naaijkens et al. 2012)	ASC-MSCs	10% FBS, 5% hPL	BC	FT	N.M	0	3–4 days	1	1	1
(Shih et al. 2011)	ASC-MSCs	10% FBS, 10% hPL	N.M.	SD	0.2 µm	C	3–4 days	2	1	1
(Crespo-Diaz et al. 2011)	ASC-MSCs and BMSCs	FBS 10%, hPL 5%	N.M.	FT	70 µm	1	N.M.	3	1	1
(Govindasamy et al. 2011)	DPSCs	10% FBS, 10% hPL	PRP	FT	0.40 µm	1	3–4 days	1	1	1
(Shichinohe et al. 2011)	BM-MSCs	10% FBS, 5% hPL	PRP	FT	N.M	N	3–4 days	2	3	1
(Xia et al. 2011)	BM-MSCs	10% FBS, 7.5% hPL	Aph	FT	0.22 µm	A−/B+	N.M.	1	2	1
(Cholewa et al. 2011)	ASC-MSCs	10% FBS, 10% hPL	N.M.	FT	N.M	1	3–4 days	1	1	1
(Abdelrazik et al. 2011)	BM-MSCs	10% FBS, 10% hPL	Aph	FT	N.M	0	3–4 days	3	1	1
(Horn et al. 2010)	BM-MSCs	10%FBS, 10% hPL	BC, Aph	FT	0.2 µm	1	3–4 days	2	2	1
(Blande et al. 2009)	ASC-MSCs	10% FBS, 2.5%, 10% hPL	N.M.	FT	0.22 µm	1	1 day	1	1	2
(Bieback et al. 2009)	BM-MSCs	10% FBS, 10% HS, 10% tPRP, 10% pHPL	PRP	Thr, FT	0.2 µm	1	3–4 days	1	1	1
(Prins et al. 2009)	BM-MSCs	10% FBS, 5% hPL	N.M.	FT	N.M	1	3–4 days	1	1	1
(Zaky et al. 2008)	BM-MSCs	10% FBS, 5% hPL	PRP	FT	N.M	1	3–4 days	1	1	3
(Schallmoser et al. 2007)	BM-MSCs	10% FBS, hPL 10%	PRP	FT	0.22 µm	1	3–4 days	1	1	1
(Capelli et al. 2007)	BM-MSCs	10% FBS, 5% hPL	PRP	FT	N.M	1	3–4 days	2	1	1
(Reinisch et al. 2007)	hUMC-MSCs	10% FBS, 10% hPL	PRP	FT	0.22 µm	1	3–4 days	2	1	1
(Doucet et al. 2005)	BM-MSCs	10% FBS, 5% hPL	Aph	FT	N.M	A	3–4 days	3	1	1

**Proliferation rate (P.I.)**: Higher in hPL (1); No difference (2); Not mentioned (3)

**Immunophenotype (CD)**: Expression CD >/= (1); No expression or < (2); Not mentioned (3)

**Morphology (MO)**: Smaller elongated spindle shape (1); No difference (2); Not mentioned (3); Different shape (4)

**Differentiation lineage (Diff. Line)**: Not mentioned (0); No difference (1); High trilineage (3); High adipogenic (A); High osteogenic (B); High chondrogenic (C); Low adipogenic and osteogenic (AB−); Low adipogenic and high osteogenic (A−/B+); Neural differentiation (N)

**hPL manufacturing**: Industrial (I); Buffy coat (BC); Platelet-rich plasma (PRP); Apheresis (Aph); Apheresis and/or Buffy Coat; Not mentioned (N.M.)

**Type of platelet lysis**: Freeze/thaw (FT); Solvent/detergent inactivation (SD); Sonification (Son); Calcium addition (Ca); Thrombin activation (Thr) and/or FT; Irradiation (UV); Solvent/detergent inactivation + irradiation and/or FT (SD + UV, FT); Solvent/detergent inactivation + Ca addition and/or FT (SD + Ca, FT); Calcium addition and/or FT (Ca, FT); Irradiation and/or FT (UV, FT); Not mentioned (N.M.)

**Type of cells**: Human umbilical cord-MSCs (hUMC-MSCs); Bone marrow-MSCs (BM-MSCs); Adipose derived-MSCs (ACS-MSCs); Human corneal stromal MSCs (hC-MSCs); Adipose derived-MSCs (ACS-MSCs) and/or human skin fibroblast (hF); Dental Pulp-MSCs (DPSCs); Adipose derived-MSCs (ACS-MSCs) and/or Bone marrow-MSCs (BM-MSCs); Not specify the source (MSCs); BM-MSCs, AT-MSCs, hUMC-MSCs, histiocytic lymphoma U-937, human chondrocyte, T-lymphocyte CD4/CD8 (Others)

## Results

The initial search process found 271 papers, which was reduced to a final selection of 56 articles, excluding reviews, studies published before 2005, articles where the FBS/FCS control were missing or when MSCs came from other sources than human tissue (Table 1). The majority of the papers (83.9%) reported a higher proliferation index for the hMSCs cultured with hPL compared to those cultured with FBS (without any distinction based on the extraction or lysate preparation methodology). In the remaining (14.3%) no differences were mentioned, while only one case lacked this information (Table 2). The evaluation of immunophenotype showed that in both FBS and hPL culture, the cells expressed similarly surface markers CD73, CD90, and CD105, in about 78.6% of the papers.

**Table 2:** Proliferation rate

Proliferation rate (P.I.)	N	%
Higher in hPL (1)	47	83.93
No difference (2)	8	14.29
Not mentioned (3)	1	1.79

A lower or lack of expression of these three CD markers was reported in 10.7% of the studies, while in another 10.7% it was not mentioned (Table 3). The differentiation capacity along the osteocyte, adipocyte, and chondrocyte lineage did not show any significant difference in the majority of the studies (57.1%). In 10.7% of reports, a lower adipogenic potential was highlighted. Just a few papers showed a higher potential for hPL-hMSCs to differentiate into only the osteocyte (10.7%), all three lineages (5.4%) or adipocyte (3.6%) or chondrogenic (1.8%) line. In one case neurogenic differentiation was obtained with a specific protocol. In the remaining 17.9% of the papers, the differentiation potential was not tested (Table 4).

**Table 3:** Immunophenotype

Immunophenotype (CD)	N	%
Expression CD >/= (1)	44	78.57
No expression or < (2)	6	10.71
Not mentioned (3)	6	10.71

**Table 4:** Differentiation lineage

Differentiation lineage (Diff. Line)	N	%
Not mentioned (0)	10	17.86
No difference (1)	32	57.14
High trilineage (3)	3	5.36
High Adipogenic (A)	2	3.57
High osteogenic (B)	1	1.79
High chondrogenic (C)	1	1.79
Low adipogenic and osteogenic (AB−)	1	1.79
Low adipogenic and high osteogenic (A−/B +)	5	8.93
Neural differentiation (N)	1	1.79

Analyzing cell morphology, the hMSCs phenotype with hPL was more often (44.64%) spindle-shaped, elongated and smaller compared to cells cultured in FBS, while in 23.2% no significant morphological differences were found. In 37.9% of articles cell morphology was not described. (Table 5).

**Table 5:** Morphology

Morphology (MO)	N	%
Smaller elongated spindle shape (1)	25	44.64
No difference (2)	13	23.21
Not mentioned (3)	17	30.36
Different shape (4)	1	1.79

hMSCs were mainly sourced from bone marrow (BM-MSCs, 42.9%) or adipose tissue (AT-MSCs, 25%). In 5.4% of the studies hMSCs isolated from both of these sources were used. Alternative sources (globally 23.2%) such as dental pulp stem cells (DPSCs), corneal stromal cells (hCMSCs) and umbilical cord mesenchymal cells (hUMCs) were also tested without relevant statistical difference between hPL and FBS additive medium, except for proliferation where, similarly to BM and AT, hPL accretes it. In one case the origin of the hMSCs was not mentioned (Table 6).

**Table 6:** Type of cell

Type of cell	N	%
Human umbilical cord-MSCs (hUMC-MSCs)	8	14.29
Bone marrow-MSCs (BM-MSCs)	24	42.86
Adipose derived-MSCs (ACS-MSCs)	14	25.00
Human corneal stromal MSCs (hC-MSCs)	2	3.57
Adipose derived-MSCs (ACS-MSCs) and/or human skin fibroblast (hF)	2	3.57
Dental Pulp-MSCs (DPSCs)	1	1.79
Adipose derived-MSCs (ACS-MSCs) and/or Bone marrow-MSCs (BM-MSCs)	3	5.36
Not specify the source (MSCs)	1	1.79
BM-MSCs, ASC-MSCs, hUMC-MSCs, histiocytic lymphoma U-937, human chondrocyte, T-lymphocyte CD4/CD8 (Others)	1	1.79

PRP (26.8%), plateletpheresis (19.6%) and buffy coat (16.1%) were the most prevalent manufacturing methods. The remaining 33.9% of papers applied an industrial preparation or did not specify the technique or origin of the hPL (Table 7). The platelets were lysed by repeated freeze/thaw cycles in 57.1% of the reports, direct platelet activation by adding thrombin was used in 3.6%, solvent/detergent (S/D) treatment in 3.6%, CaCl_2_ addition (1.8%) and sonification in 1 case (1.8%). Sterilisation of hPL by irradiation of the platelet lysate was described in 7.1% of the cases. A combination of these different techniques with UV treatment was described in 2 out of 56 cases (3.6%) with freeze/thaw (F/T) and one associated with solvent/detergent (S/D) treatment (1.8%). Finally, a S/D treatment plus calcium addition followed by F/T was mentioned in one case (1.8%). Sterilisation methods were not reported in 19.6% of publications (Table 8).

**Table 7:** hPL manufacturing

hPL manufacturing	N	%
Industrial (I)	9	16.07
Buffy coat (BC)	9	16.07
Platelet-rich plasma (PRP)	15	26.79
Apheresis (Aph)	11	19.64
Apheresis and/or Buffy Coat	2	3.57
Not mentioned (N.M.)	10	17.86

**Table 8:** Type of platelet lysis

Type of platelet lysis	N	%
Freeze/thaw (FT)	32	57.14
Solvent/detergent inactivation (SD)	2	3.57
Sonification (Son)	1	1.79
Calcium addition (Ca)	1	1.79
Thrombin activation (Thr) and/or FT	2	3.57
Irradiation (UV)	1	1.79
solvent/detergent inactivation + irradiation and/or FT (SD + UV, FT)	1	1.79
solvent/detergent inactivation + Ca addition and/or FT (SD + Ca, FT)	1	1.79
Calcium addition and/or FT (Ca, FT)	2	3.57
Irradiation and/or FT (UV, FT)	2	3.57
Not mentioned (N.M.)	11	19.64

Overall, 54%of the studies did not mention the type of filter used, while 32% applied a 0.2 µm, 2 out of 56 papers a 0.4 µm, 3 articles a 70 µm or a combination of 0.4/0.2 µm (2) and 0.2/0.1 µm (1) (Table 9).

**Table 9:** Filter

Filter	N	%
0.2 µm	18	32.14
0.4 µm	2	3.57
70 µm	3	5.36
0.2/0.1 µm	1	1.79
0.4/0.2 µm	2	3.57
N.M.	30	53.57

The heterogeneity of the protocols used to prepare hPL further increased, with the inclusion of thrombin or heparin in the suspension or additional extraction conditions (centrifugation and additional filtration steps).

In Table 10 the main growth factors and protein composition of the hPL are listed. Compared to FBS the concentration of PDGF-AA, -AB, -BB, TGF-Beta, VEGF and IGF-1 is higher in hPL, while the total protein and albumin values are respectively lower or the same.

**Table 10:** Composition of human platelet lysate reported in literature: growth factors and proteins

First author	Year	Estimated?	Type of Medium	PDGF (ng/ml)	PDGF-AA (ng/ml)	PDGF-AB (ng/ml)	PDGF-BB (ng/mL)	TGF-Beta (ng/ml)	VEGF (ng/ml)	bFGF (ng/ml)	EGF (ng/ml)	BDNF (ng/ml)	IGF-1 (ng/ml)	HGF (ng/ml)	Beta-NFG	Protein Tot (mg/ml)	Albumin (mg/ml)
Barro	2019	Y	hPL			80.00		32.00	0.17	0.40	3.80	100.00	80.00	0.13		58.32	41.00
Barro	2019	Y	I-hPL			125.00		33.00	0.40	0.42	5.90	80.00	80.00	0.25		26.85	17.00
Barro	2019	N	DVI-hPL			8.16		35.17	0.31	0.35	4.70	25.73	35.17	0.24		26.27	16.00
Chen	2018	N	SDPL			45.00	11	75.00	0.40	0.55	0.85	20.00	17.50	0.12		56.00	
Chen	2018	N	FTPL			45.00	11	75.00	0.40	0.55	0.85	20.00	17.50	0.12		56.00	
Chen	2018	N	GBPL			45.00	11	75.00	0.40	0.55	0.85	20.00	17.50	0.12		56.00	
Chen	2018	N	GBHPL			45.00	11	75.00	0.40	0.55	0.85	20.00	17.50	0.12		56.00	
Mangum	2017	N	hPL		4.57	17.84	5.24	5.5	0.75	5.5	0.5	5.5		0.5			
Bernardi M	2017	N	hPL		0.00	0.03	0.01	0.05	0.00	0.00	0.00		0.00				
Bernardi M	2017	N	PR-SRGF		0.01	0.14	0.01	0.04	0.00	0.00	0.00		0.11				
Pierce	2017	N	hPL				10.70		0.80	0.20	3.40					54.00	
Viau	2017	N	PR-hPL			28.78		55.20	0.65	0.15	1.48		32.55				
Viau	2017	N	hPL			28.78		55.20	0.65	0.15	1.48		32.55				
Escobar	2016	N	hPL				0.42	0.15	0.00	0.30	0.42		0.00				
Shirzad	2016	N	UCB-PL			577.97		56.04		0.06			501.62			90.46	
Shirzad	2016	N	PB-PL			391.62		29.31		0.06			325.50			49.14	
Suri	2016	Y	hPL	70.00				250.00	0.90		0.20						
Suri	2016	Y	PhPL	40.00				210.00	1.20		0.27						
Witzeneder	2013	N	hPL p				18.11	32.74		0.36	0.01		33.14			61.20	
Witzeneder	2013	N	hPL n				22.14	54.86		0.17	0.01		4.42			7.70	
Witzeneder	2013	N	huS				2.38	4.89		0.00	0.01		85.98			66.60	
Mojica-Henshaw	2013	N	hPLFreeze/thaw -196/4 °C		21.00	50.00	13.90			0.19	4.40					58.64	
Mojica-Henshaw	2013	N	hPL Freeze/thaw -80/4 °C		13.70	43.80	8.70			0.16	3.60						16.12
Shanskii	2013	N	hPL		6.91	3.98	8.20	16.30	0.15				108.70			26.00	42.50
Fekete	2011	N	PL-PCC		239.41	571.73		139.03	0.33	0.50							
Crespo-Diaz	2011	N	hPL	10.00				125.00	0.45	0.20	20.00		130.00				
Shichinoche	2011	N	hPL				0.14	2.85				0.79		0.00	0.00		
Xia	2011	N	hPL			0.00		5.15		0.04			0.00				
Schallmoser	2007	N	hPL		7.42	40.46			0.00	0.06	0.19						
Doucet	2005	N	hPL			30.89		50.84		0.09			106.67				
Doucet	2005	N	PPP			0.24		0.51		0.00			107.41				
Mean Values				40.00	36.63	99.06	7.50	57.24	0.40	0.23	2.77	35.82	78.81	0.13	0.00	49.95	26.52
Shanskii	2013	N	FBS		0.14	0.03	0.00	0.85	0.00				64.10			58.64	34.45
Shichinoche	2011	N	FBS				0.14	1.27				0.00		0.00	0.00		
Doucet	2005	N	FCS			0.00		6.21		0.00			72.82				
Mean Values					0.14	0.01	0.07	2.78	0.00	0.00		0.00	68.46	0.00	0.00	58.64	34.45
Shichinoche	2011	N	FBS-BMSCs cultures				0.00	1.73				0.03		1.72	0.00		
Shichinoche	2011	N	hPL-BMSCs cultures				0.00	3.84				0.05		2.55	0.00		

However, in the majority of the studies the analysis of the growth factors composition was limited to compare the different type of hPL lysate preparations, instead of analysing the variation between FBS and hPL. No significant variation in GF composition was discovered that related to platelet lysate manufacturing methods.

## Discussion

Currently, there is no consensus in the literature regarding the most appropriate medium additives for in vitro expansion of hMSCs in a clinical setting. According to our selection criteria, all included publications analyzed hMSCs morphology, proliferation, immunophenotype and mesodermal differentiation potential, allowing the effects of hPL or FBS supplement on these parameters to be compared. Most studies, assessed here, used hMSCs derived from bone marrow or adipose tissue.

This lack of consensus can be explained by the intrinsic variability and complexity of both FBS and hPL: both their composition is not completely defined in terms of protein, cytokine and elementary components and, moreover, hPL manufacturing is not a standardized process. All these factors increase the challenge to judge the respective usefulness of these media supplements.

Despite these differences, the majority of the authors agreed that media supplemented with hPL support a higher proliferation index compared to FBS supplemented ones or other, serum-free media [3, 9, 10, 13, 15–17, 19, 24, 31, 33, 35, 37–65].

A conceivable explanation of this data has to be found in the composition of hPL; platelet alfa-granules contain coagulation factors, adhesion molecules, protease inhibitors, bFGF, EGF, HGF, VEGF, IGF-1, TGF-ß, PDGF-AA, PDGF-AB, PDGF-BB and a variety of cytokines and chemokines [66, 67]. Their respective role in conveying the increase in cell growth and proliferation rates and thus cell expansion has not yet been completely resolved. One probable cause could be the known effect of some of these growth factors to increase cell proliferation (particularly PDFG-BB and bFGF) [67].

Specifically, three studies compared the growth factor concentration in both FBS and hPL showing that in FBS the following growth factors are present in lower quantity than in hPL (mean values respectively for FBS and hPL in ng/mL): PDGF-AA (0.14, 36.63), PDGF-AB (0.01, 99.06), PDGF-BB (0.07, 7.50), TGF-ß (2.78, 57.24), bFGF (0.0, 0.23), VEGF (0.0, 0.40), (IGF-1) (68.46, 78.81) and (BDNF) (0.0, 35.82) (Table 2) [19, 55, 68].

In about half of the publications the authors specifically mentioned that MSCs expanded in hPL showed a different morphology, compared to those grown with FBS; they were smaller, more elongated and more often spindle shaped. In the remaining publications, the authors did not investigate or did not mention any change in cellular morphology. Only one publications reported hMSCs to be larger when cultured with hPL [46].

In the vast majority of reports the ability of hMSCs to be differentiated along pre-determined lineages was not affected by being cultured using hPL: adipogenic, osteogenic and chondrogenic differentiation were demonstrated with no relevant difference in effectiveness or potential to the same hMSCs cultured with FBS. In three cases MSCs, expanded in hPL, were more effectively differentiated along osteogenic, chondrogenic and adipogenic routes [37, 49, 58]; others reported a difference only for osteogenic [17, 40, 42, 47, 54] or adipogenic differentiation respectively [19, 31]. Only one publication reported that FBS supported osteogenic and adipogenic differentiation to a higher degree [57]. A single study investigated neural differentiation of BM-MSCs (growth media containing 10% FCS and 5% hPL respectively), reporting no significant differences in terms of proliferation and differentiation potential [68].

The BC and PRP techniques, that were applied to obtain the pooled platelets, were not distinguished in how they affect hMSCs grown in hPL. Because some authors compared FBS with industrial hPL products, which were prepared using undisclosed methods of isolation and lysis, we could not draw any conclusion of the influence of hPL isolation and preparation methods on cells.

The release of platelet content to create hPL can be achieved by a variety of methods: freeze and thaw, activation by either thrombin or calcium, solvent/detergent treatment and sonification. In summary of the assembled data from the publications reviewed here, the technique by which lysis was achieved seems to have no significant influence on MSCs growth, proliferation or differentiation potential [13, 20, 31, 33, 37, 40, 45, 54, 66, 69].

The risk of biological contamination of hPL could be minimized by adapting manufacturing methods to include additional steps such as detergent, UV irradiation, or irradiation in combination with psoralen. Shih illustrated how inactivating hPL by solvent/detergent treatment did not affect hMSCs proliferation, lineage differentiation capability and CD marker expression compared to either FBS or hPL control [70]. In the same way photoactivation with UV light or psoralen did not alter BM-MSCs growth or CD markers of stemness [71, 72].

Further studies are needed as the different techniques used for hPL generation could conceivably interfere with its composition, for instance, the quality and quantity of growth factors and cytokines.

Despite the promising data which showed that hPL can be an excellent alternative to FBS, open questions remain regarding quality and production. At this time, according to our findings, not enough information is available due to a lack of universally accepted international guidelines for hPL preparation and analysis. Recently, in response to similar results reported elsewhere, a collaboration between the American Association of Blood Banks (AABB) and the International Society of Cell Therapy was initiated to address hPL quality control and standardize hPL manufacturing [30, 73].

## Limitations

The data, in the selected literature revised, showed a large variety in terms of definitions e.g. the proliferation index, as we named it, incorporates any measurements related to cell proliferation such as repeated cell counts, population doubling time (PDT), cumulative PDT, generation time, number of colony forming unit fibroblast (CFU-F); the methods and values varied considerably between studies, made the comparison difficult and sometimes even impossible to arrive at any meaningful conclusion.

Moreover, the methodology of hPL manufacturing, as previously highlighted, encompassed a wide range of parameters, starting with the heterogenicity of blood sample management, the extraction protocols, such as BC or PRP, and the multiple platelet lysis strategies. Furthermore, almost each publication mentioned additional steps that deviated from the generalised protocol e.g. additional centrifugation, filtration, thrombin or heparin addition, suspension in different intermediate solution or irradiation steps. All these aspects somewhat limited our ability to combine the data in a meaningful way.

For future works we strongly recommend to the authors: (A) include FBS controls (lead to the exclusion of 11 publications after a further selection of 271 records); (B) provide a detailed preparation methodology for hPL; (C) measure the growth factor, cytokine and micronutrient content; (D) include a clear characterization of hMSCs phenotype including morphology, CD marker profile and trilineage differentiation capability.

Finally, the proliferation index must be comparable: researchers use a wide range of methods such as repeat cell counts, population doubling time, cumulative population doubling time, generation time, MTT or Alamar blue and number of colony forming unit-fibroblasts (CFU-F) to evaluate the cell growth, which reduces the ability to compare the respective data.

## Conclusion and future perspectives

In the literature reviewed, no consensus was expressed in terms of the source of the medium supplement (FBS/hPL), neither a standard procedure of hPL manufacturing clearly emerged. The quality of the final product, in terms of different impact on hMSCs biology, may be significantly influenced by the production methods utilized.

Despite this heterogeneity in hPL manufacturing, the majority of the publications agreed that hPL was at least as effective as FBS with regards to maintaining hMSCs proliferation, immunophenotype and differentiation capacity.

According to the international society of cellular therapy (ISCT) and the international fat applied technology society (IFATS) which have demanded specific guidelines for the characterization and selection of ADSCs in regenerative medicine, hPL could be considered an alternative to the use of FBS in hMSCs culture—especially with a view to their clinical use.

Based on our review we envisage that hPL research should address a standardization of hPL production process. hPL should ideally come from a limit number of pooled PL (platelet) units in order to minimize the risk of patient-to-patient disease transmission but guarantee the highest levels of GFs in the final product. The samples have to be tested for a standardized set of bacterial, yeast, fungal and viral contamination and PL donors have to be excluded in case of a potential anamnesis exposure.

It is necessary to define a minimal concentration for the main components due to be sure that the final product can support MSCs adequately, independently of the different manufacturing methods or lysis or pathogen inactivation.

Reaching the minimum content requirements, to reduce costs and preserve the limited sources of PL, a blended mixture with serum free and lysate could be speculated to obtain an ideal growth cell environment.

Studies which compared different PL extraction with same lysis process or the variety of lysis techniques from the same isolation strategy, are needed to understand the most efficient combination in terms of GFs and protein content. This could address to a standardization of the methods to obtain a more comparable hPL.

## Data Availability

Yes.
